# The Role of Lipid Subcomponents in the Development of Atherosclerotic Plaques

**DOI:** 10.31083/j.rcm2405139

**Published:** 2023-05-05

**Authors:** Lei Nie, Xuying Xiang, Cheng Wen, Feng Zhang, Yuanpeng Xia, Yong Wang, Ling Mao

**Affiliations:** ^1^Department of Neurology, Union Hospital, Tongji Medical College, Huazhong University of Science and Technology, 430022 Wuhan, Hubei, China

**Keywords:** atherosclerosis plaque, lipid metabolism, lipid subcomponents

## Abstract

Atherosclerosis (AS) is a long-standing cardiovascular and cerebrovascular disease. Its occurrence and development are related to the pathophysiology of lipids including cholesteryl ester (CE), cholesterol, triacylglycerol (TG), and phospholipid (PL). In this review, we focus on the roles and possible mechanisms of different lipid subcomponents in the process of AS, and provide new ideas for the prevention, diagnosis and treatment of AS.

## 1. Introduction

Atherosclerosis (AS) is a disease that causes plaque formation in the arterial lining. It remains the largest cause of death globally [1]. A major driver of atherosclerotic plaque initiation is the progressive accumulation of lipids, which are derived from circulating lipids, at the sites of endothelial dysfunction in the arterial wall [2]. The influx of lipids and their subsequent alteration in the arterial wall induce an inflammatory response that exacerbates the atherosclerotic process [3, 4]. In this review, we will discuss the differences in the content of different lipids at distinct lesion locations, the effects of different lipid components on the overall progression of plaques, and the effects and mechanisms of various lipids in various plaque-forming cells.

## 2. Mainstream Lipid Detection Methods

The conventional method for detecting lipids in plaques is to uniformly extract the lipids in plaques with various organic solvents, and then react with enzymes and other reagents to separate or separate them based on their varied physicochemical properties of various components in the lipids. It is decomposition, and its corresponding extract or decomposition product, that is quantified in order to assess, its original content and proportion in the plaque. For example, Lawrence and Robert utilized acetone and chloroform/methanol (2/1, v/v) to analyze myocardial lipid profiles of aortic tissue extracted from various plaque regions [5]. Cholesterol and fatty acids can be directly measured, while cholesteryl esters (CE) and phospholipids (PL) must first be decomposed into products by relevant hydrolase and then measured. Through this method, we can tell that, in general, CE is the predominant constituent of atherosclerotic plaques, followed by PL, free cholesterol (FC), and triacylglycerol (TG) [6].

This form of chemical assay can directly measure the lipid content in plaques, and at the same time, the corresponding lipid subcomponents may be extracted for future research. But unfortunately, due to its complex operation and the inconvenient nature of analyzing highly subdivided lipid subcomponents, this technique is gradually being supplanted by other methods.

In recent years, Mass Spectrometry Imaging has replaced chemical detection as the method of choice for determining plaque lipid composition. The main reason for this shift is that it is very challenging for chemical detection methods to precisely measure the lipid components in more subdivided categories of lipid components with a limited sample size, thus limiting our, understanding of the specific lipid metabolism in plaques. Matrix-assisted laser desorption/ionization mass spectrometry imaging (MALDI-MSI) enables the detection of differences in plaque lipid composition, including sphingomyelin and lysolecithin [7, 8]. More importantly, this method quantifies the more subdivided lipid subcomponents by retaining the plaque as close to the body as feasible without manipulating plaque components. This provides the possibility to determine the variances in lipid subcomponents of plaques at different sites and the stability within the plaque. This procedure only measures the different mass-to-charge ratios of lipids, and what subcomponents of the corresponding lipids are to be identified after extraction. Therefore, numerous studies can only describe unnamed lipids. There are differences in the quality and sub-components of lipids. Therefore, research involving the extraction of lipid components is still a challenging topic.

## 3. Differential Trends of Lipid Content in Plaques

By quantitatively analyzing lipid plaque composition, Lawrence and Robert found the various characteristics of individual plaques. They counted the data of different parts of the plaque obtained by chemical analysis [5]. It has been determined that in plaques, CE content is the highest, followed by PL, third FC, and triglyceride (TG). The content of CE and FC increases the closer they are to the lipid core, but the content of PL increases in the opposite direction, and is even more abundant than that of CE towards the border of the plaque. TG did not exhibit a more obvious dispersion pattern.

In addition, not only the lipid content of different parts of the same plaque is different, but also the lipid content of different stable plaques. Previous studies have separated the resulting plaques into two groups by stability and measured lipid subcomponents using mass spectrometry. It was discovered that the lipid content of unstable plaques was higher than that of stable plaques. CE and FC accounted for a greater share, especially the proportion of FC, suggesting it may be responsible for plaque instability. The more detailed measurement of mass spectrometry allowed us to find statistically significant differences in many lipid subcomponents (Table 1, Ref. [6, 7, 8]). Table 1 shows the lipid subcomponents with differential distribution in different studies. Many lipids have not been purified by chemical methods to analyze their specific molecular composition, only their mass-to-charge ratio (m/z value) is used for differentiation.

**Table 1.** **Differences in lipid subcomponents of stable and unstable plaques**.

	Lipid most abundant in unstable plaques	Lipid most abundant in stable plaques
CE	CE(10:0),CE(14:0),CE(18:0),CE(18:2),CE(18:3),CE(20:0),CE(20:1),CE(20:2),CE(20:5),CE(22:3),CE(22:4)	CE(18:1),CE(20:3),CE(20:4),CE(22:5),CE(22:6)
PL	SM(d18:1/15:0),IPC(18:0),IPC(20:2),PC(32:1),PC(44:12),DG(34:1),DG(36:2)	PC(34:1),PC(36:4),PC(38:4)
TG	TG(52:2)	

It is already known that there are differences in lipid composition between different portions of the plaque, and there are differences in lipid composition between stable and unstable plaques. These differences in the lipid subcomponents were found by mass spectrometry [6, 7, 8]. SM, sphingomyelin; IPC, inositolphosphorylceramide; PC, phosphatidyl choline; DG, diacylglycerol.

## 4. Lipid Metabolism during Macroscopic Plaque Progression

### 4.1 Cholesteryl Ester (CE) and Free Cholesterol (FC)

Cholesterol is a derivative of cyclopentane polyhydrophenanthrene. The chemical formula is C27H46O. It is a white or pale yellow crystal that is the primary steroid compound in the human body. It is an essential component of cell membranes. Lipoproteins in plasma are also rich in cholesterol; and 70% of them form CEs with long-chain fatty acids. Intracellular FC is catalyzed by fatty acylcholesterol acyltransferase (ACAT) to generate CEs. The FC in plasma is catalyzed by lecithin cholesterol acyltransferase (LCAT) to generate CEs and lysophosphatidylcholine (LPC) [9]. CE is the most abundant lipid in plaques, and is the dominant lipid in the core of the plaque [10]. FC is the third most abundant lipid and its concentration drops steadily as it approaches the core of the plaque. This suggests that there is a conversion relationship between CE and FC in the process of plaque formation.

During plaque formation, low-density lipoprotein cholesterol (LDL-C), in particular oxidatively modified LDL-C (ox-LDL-C), accumulates in large amounts in the lesion, resulting in the accumulation of FC and CEs in the arterial wall, and result in acute coronary syndromes [11]. Studies have indicated that the interconversion between FC and CEs affects the plaque stability. Under certain conditions, neutral cholesteryl ester hydrolase (NCEH) in foam cells found in plaques can hydrolyzes CE to FC, which is then effluxed [12]. After CE is converted to FC, it can be effluxed from the foam cells, thus reversing the intra-plaque accumulation of lipids, and stabilizes the plaque. The excess FC can be converted into CE by acyl-Coenzyme A acetyltransferase 1 (ACAT1) and stored again. The metabolic disorder affecting foam cells causes excessive conversion of FC and difficulty in its outflow from foam cells. The cytotoxicity of FC leads to further collapse of foam cells. FC persists in significant quantities in the extracellular matrix as crystals, which lowers plaque stability and worsens the prognosis [13]. Based on these findings, cholesteryl ester hydrolase (CEH) and ACAT1 have become popular therapeutic targets for AS.

### 4.2 Triglyceride (Tg)

Tgs are synthesized by the esterification of glycerol and fatty acids, and are stored in the body in an anhydrous state. It is the energy substance with the largest storage and production capacity in humans. Similar to cholesterol and low-density lipoproteins, Tgs play a role in AS progression in the form of triglyceride-rich lipoproteins (TRLs). TRL is the collective name for chylomicron (CM) and very low-density lipoprotein (VLDL). Its effect on plaques has been validated by recent advances in human genetics, as well as by numerous epidemiological, preclinical, and clinical trial results [14]. The Tg deposited in the plaque by TRL is the fourth most abundant lipid, and its relative content decreases as it is closer to the center of the plaque. This tendency is more likely a result of the relative change induced by the increase in CE, for which there are no published data.

Other studies have suggested that, unlike cholesterol’s dual direct role as a lipid core affecting plaque stability and a metabolite interfering with foam cells to affect plaque lipid deposition, TG plays a more indirect role by affecting cholesterol metabolism; thus, affecting the progression of the disease [15, 16, 17]. In addition, there are few studies on the independent effect of TG on plaque stability, given that its concentration is rather low. However, this does not imply that TG research is irrelevant to the diagnosis and treatment of AS. Many studies have confirmed that, excluding the influence of plasma cholesterol levels, plasma TG levels are still positively correlated with the progression of AS [18], and they should be routinely monitored. Intervention is still required for patients with cardiovascular and cerebrovascular disease whose TG levels do not meet the guideliness. Medications, such as fibrates, that mainly lower the TG levels can also reduce the incidence of cardiovascular and cerebrovascular disorders.

### 4.3 Phospholipid (PL)

PLs are complex lipids containing phosphoric acid that are an important component of biological membranes. PI is also plentiful in plaques, and surpasses cholesterol as the main lipid away from the plaque core, and gradually decreases as it approaches the core. According to their main chain structures, they are divided into phosphoglycerolipids and sphingomyelins. The most abundant phosphoglycerolipids in the human body are phosphatidylcholine (lecithin) and phosphatidylethanolamine (cephalin). Among the phosphoglycerolipids, phosphatidylcholine and phosphatidylethanolamine are most abundant in plaques and plasma. More notably, LPC and lysophosphatidylethanolamine (LPE), which are produced by the hydrolysis of phosphoglycerolipids by phospholipase A2, are considered to be a novel class of atherosclerotic vascular indicators.

Sphingomyelin is a PL composed of sphingosine or dihydrosphingosine, and its molecule does not contain glycerol. It is a fatty acid molecule that is linked to the amino group of sphingosine through an amide bond. Sphingomyelin is the most abundant sphingolipid in the human body, and it is catalyzed by sphingosine acyltransferase to generate ceramide (also known as ceramide synthase, CerS). Circulating levels of ceramides have been found to be positively correlated with the s of AS [19].

Studies have shown that compared with young ApoE-/- mice and wild-type mice, aged ApoE-/- mice have significantly more LPC and LPE in the aortic atherosclerotic plaques [20]. The FC can be catalyzed by LCAT to generate CE and lysolecithin [9], which suggests that LPC, a by-product of the reaction, may influence the mutual conversion of FC and CE and potentially affect the stability of plaques. All of these findings suggest that lysophospholipids, including LPC and LPE, may play a significant role in advanced AS.

Sphingomyelin is a PL containing either sphingosine or dihydrosphingosine and its molecule does not contain glycerol; it is a molecule of fatty acid linked to the amino group of sphingosine by an amide bond. A study utilizing mass spectrometry imaging to evaluate the composition of advanced atherosclerotic plaques has revealed that sphingomyelin and oxidized CEs are enhanced exclusively in the necrotic intimal region [21]. Inhibition of endogenous sphingomyelin synthesis reduces the atherosclerotic plaque size in rodents [22], suggesting its role in the formation and progression of AS. Sphingomyelin is the most abundant sphingolipid in the human body, which is catalyzed by sphingosine acyltransferase (also known as ceramide synthase, CerS) to produce ceramide. Circulating levels of ceramides have been shown to be positively correlated with the degree of AS [23]. Studies have suggested ceramides serve as a biomarker to distinguish between peripheral arterial disease and stable coronary artery disease [24]. Both are AS-based diseases and have no obvious clinical manifestations, but the former has a higher incidence of cardiovascular and cerebrovascular events. These studies have shown that sphingomyelin and its metabolite ceramide have the potential to become a new biochemical marker of AS, as well as a possible therapeutic for improving the prognosis in AS.

### 4.4 Fatty Acids

Among the lipids listed above, fatty acids also play a hidden role in AS progression. Fatty acids are frequently mixed with other chemicals to form complexes involved in biological activities. They combine with cholesterol to form CEs; and are also the primary constituents of Tgs and PLs. The role of non-ester-forming free fatty acids in plaques has been rarely reported. These lipids combine with other fatty acids to form different lipid subcomponents, which play a role in the AS process. There are many different classification methods of fatty acids. In AS, we often divide them into two categories according to the difference between saturated and unsaturated hydrocarbon chains, namely: saturated fatty acids (SFA), where there is no unsaturated bond in the hydrocarbon chain, whereas unsaturated fatty acids (UFA) have one or more unsaturated bonds in the hydrocarbon chain.

The separation and purification of these more subdivided lipid subcomponents in plaques is extremely difficult, and the involvement of the identical type of lipid components formed by different fatty acids in plaques requires further studies. In previous studies [5, 6, 7, 8] of fatty acids in plaques, the fatty acids were extracted by a hydrolysis reaction alone for analysis and research. The fatty acids (or fatty acid esters, which were equivalent to fatty acids after hydrolysis) were adjusted separately for experiments.

Although objections to the classic view regarding whether SFA is a risk factor for cardiovascular and cerebrovascular diseases have been published in recent years, the academic community remains largely in agreement that reducing the intake of SFA and replacing it with UFA can effectively reduce the occurrence of cardiovascular and cerebrovascular diseases [25]. This is based on the following widely recognized phenomenon: reduction of SFA reduces total serum levels, especially LDL-C, a key risk factor for cardiovascular disease, and therefore the greatest benefit can be obtained by replacing SFA with UFA [26].

The academic community has also offered other innovative discoveries that support the aforementioned position: a Chinese-led research team at Columbia University discovered palmitate (a common SFA) through advanced vibrational imaging technology, namely stimulated Raman scattering microscopy. SFA can facilitate the separation of solid-like domains from the endoplasmic reticulum membrane, which may be a homogenous fluid. The molecular structure of SFA is stiff and inelastic [27]. If the cell uses a considerable amount of SFAs to construct the cell membrane, it will solidify the cell membrane, which can flow freely like water, to form an isolated “island”, which will cause failure of some of the cell’s physiological activities. If the above process occurs in foam cells, it will disrupt the lipid metabolism within the plaque and trigger further plaque progression.

Another role of fatty acids in plaque formation is that serum total non-esterified fatty acids (or free fatty acids) indicate the degree of esterification of circulating lipids. There is a favorable correlation between the content of free fatty acids and the intima-media thickness of the common carotid artery [28], suggesting a new biomarker of arterial disease.

## 5. Effects of Lipids on Cells Involved in Plaque Formation

Despite the fact that there are many controversies regarding the initiation and progression of AS, there are consensus agreements on the pathophysiology of AS [29]. The intima is the deepest layer of the blood vessel wall where atherosclerotic plaques originate. In the early stage of the disease, it is still debatable whether LDL is deposited in the intima first causing local inflammation, leading to the destruction of intimal function and triggering subsequent reactions, or whether the intima is first damaged, followed by LDL deposition at the damaged site and subsequent reactions. Unprotected by plasma antioxidants, LDL particles can undergo oxidation and other modifications that promote inflammation and immunogenicity. Typical monocytes exhibit pro-inflammatory functions and then enter the intima. Monocytes circulate in the bloodstream and can adhere to molecules expressed by activated endothelial cells. Chemokines enhance the migration of bound monocytes into the arterial wall. Once monocytes have entered the intima, they can mature into macrophages and acquire features associated with the reparative or less pro-inflammatory monocyte/macrophage populations. These cells express clearance receptors that allow them to bind lipoprotein particles and transform into foam cells. Although T lymphocytes are less numerous than monocytes, they can also infiltrate the intima and regulate the function of innate immune cells, endothelial cells, and smooth muscle cells. Smooth muscle cells in the media can migrate to the intima under the action of mediators formed by the accumulation of leukocytes. The smooth muscle cell chemotactic platelet-derived growth factor generated by macrophages and deposited by activated platelets at sites of endothelial rupture or intraplaque hemorrhage, may be involved in the directed migration of medial smooth muscle cells to the intima.

These vascular smooth muscle cells (VSMCs) undergo phenotypic transformation under the action of various factors, including chemokines. They transfrom contractile types rich in contractile proteins, such as α-smooth muscle actin, to secretory types rich in other macrophage markers, such as CD68. Secretory VSMCs secrete an extracellular matrix (ECM) rich in proteoglycans and glycosaminoglycans (hyaluronic acid), in which numerous lipids are retained. Both secretory VSMCs and macrophages have an increased number of scavenger receptors (SR) during the development of the disease, and they breach the lipid metabolism balance mechanism of normal cells and are filled with lipids; thus, they generate foam cells and accelerate the progression of lesions. As the lesions progress, VSMCs and macrophages can undergo cell death, including apoptosis. Necrotic cells and debris of dying cells build up to form a necrotic, lipid-rich atherosclerotic core. Impaired excretory cell function (removal of dead cells) promotes the formation of necrotic cores.

Ultimately, the above process leads to the formation of atherosclerotic plaques. It is evident that lipids mostly interfere with the normal cellular activities of endothelial cells, macrophages, and VSMCs, thereby impacting the occurrence and development of plaques. Specific responses of each cell type to different lipids will be analyzed individually in the following sections.

### 5.1 Endothelial Cell Injury Mechanism Dominated by Lipid

**Fig. 1.**
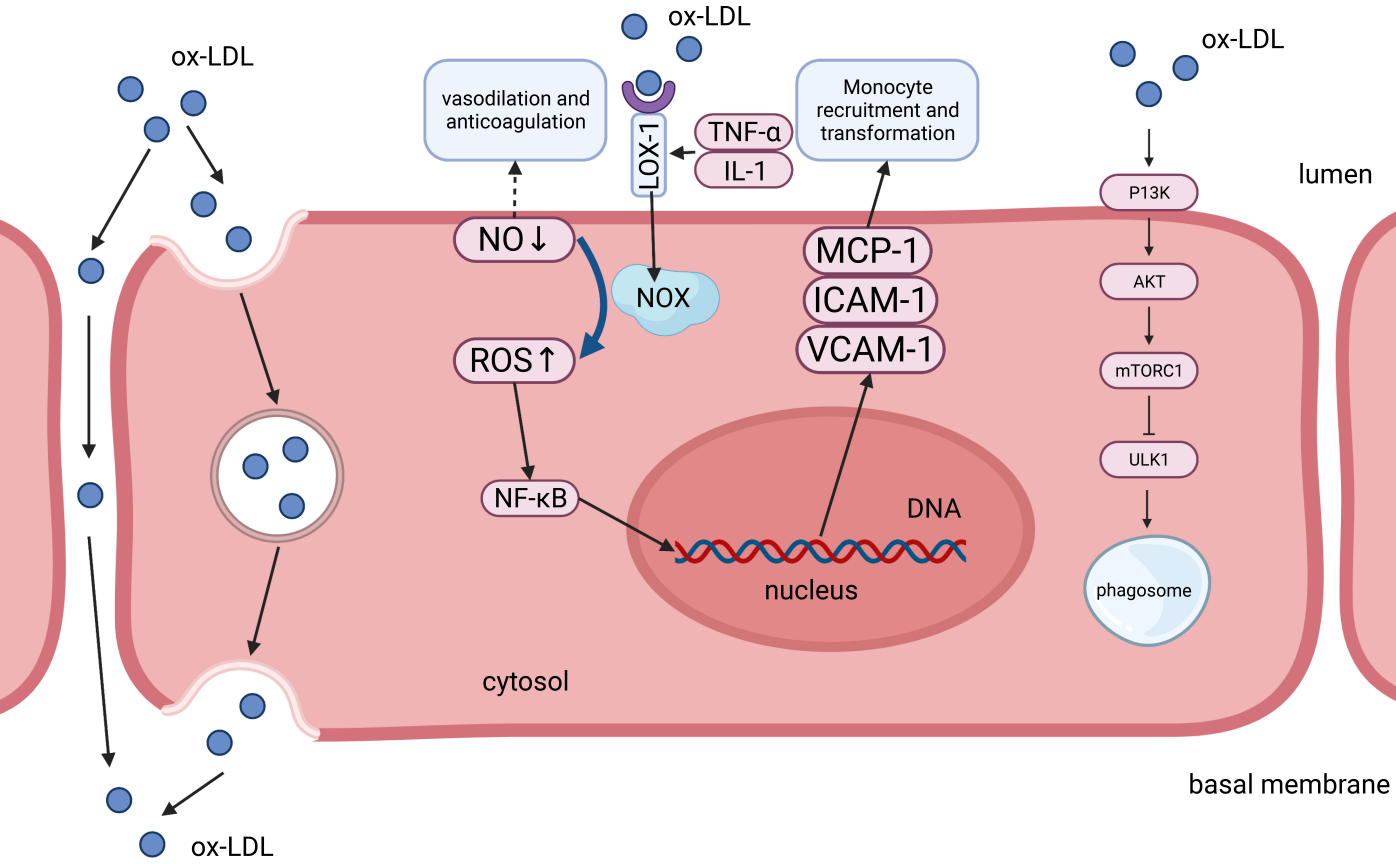
**Endothelial injury mechanism and transendothelial transport of oxidized lipoprotein**. The expression of LOX-1 is enhanced with an increase in TNF-α, IL-1, and ox-LDL levels. LOX-1 paired with ox-LDL directly generates superoxide anion ROS, and LPC, an important component of ox-LDL, and it can also boost the NOX activity and promote the conversion of NO to ROS. The increased ROS stimulates the expression of associated target genes, such as *MCP-1*, *ICAM-1*, and *VCAM-1*, through the NF-κB pathway and ultimately enhances monocyte recruitment and pro-transformation function of endothelial cells. In contrast, decreased NO has weakened vasodilatory and anticoagulant effects, which is manifested as weakened anti-atherosclerotic function of endothelial cells. Through the P13K/AKT/mTORC1 pathway, ox-LDL inhibits the key regulator of autophagy ULK1, attenuates the autophagy of endothelial cells, and promotes the development of local lesions. Oxidized lipoproteins can cross the endothelium monolayer by convection and/or diffusion between neighboring cells (paracellular leak) or transcytosis via individual cells. Transcytosis may be receptor-mediated or may occur by fluid-phase pinocytosis; it is also possible that transcellular channels may contribute to this process. By this mechanism, oxidized lipoproteins can permeate the basal membrane and participate in the subsequent development of plaques.

Regardless of the controversy around the etiology of AS, it appears that lipid damages endothelial cells. Among the various mechanisms that play a significant role in this process, the most well-known mechanism is the lectin-like oxidized low-density lipoprotein (LDL) receptor-1 (LOX-1), which was originally identified as the endothelial receptor for oxidized low-density lipoprotein (ox-LDL). The expression of LOX-1 is often regulated by cytokines, including tumor necrosis factor-α and interleukin-1 [30]. Directly related to lipids, it has been demonstrated that ox-LDL and its principal plaque-causing component LPC can significantly upregulate LOX-1 expression *in vitro * [31]. LOX-1-mediated ox-LDL acts on vascular endothelial cells, impairing their normal anti-atherosclerotic function, enhancing their ability to recruit monocytes, and inducing foam cell formation (Fig. 1).

Upon recognition, ox-LDL activates the LOX-1 extracellular lectin domain, which can either be internalized by endocytosis or phagocytosis, or remain attached to the cell surface. ox-LDL combines with LOX-1 in endothelial cells to generate superoxide anion, such as reactive oxygen species (ROS), reduce nitric oxide (NO), and diminish its anti-atherosclerotic effects, such as vasodilation and anticoagulation [32]. In addition, LPC can also increase the endothelial nicotinamide adenine dinucleotide phosphate (NADPH) oxidase (NOX) activity and the production of ROS, which ultimately attenuates endothelial cell anti-atherosclerotic function [33]. LOX-1 also activates nuclear factor kappa B (NF-κB), resulting in gene expression (chemokines and adhesion molecules) and cellular phenotype (activation or apoptosis) alterations. Chemokines and adhesion molecules are involved in the recruitment of monocytes. Monocyte chemoattractant protein-1 (MCP-1) is a monocyte chemoattractant protein. Incubation of endothelial cells with ox-LDL fundamentally triggers MCP-1 expression and monocyte adhesion to endothelial cells [34, 35]. In conclusion, following ox-LDL internalization, LOX-1 begins a vicious cycle characterized by the activation of pro-inflammatory signaling pathways, which subsequently advances an extended responsive oxygen arrangement and secretion of pro-inflammatory cytokines, with endothelial dysfunction and marked monocytes. The cell recruits an energy boost and hence promotes the phenotypic switch [36] (Fig. 1). It is already known that there are differences in lipid composition between different portions of the plaque, and there are differences in lipid composition between stable and unstable plaques. These parameters are the subcomponents of lipids for which mass spectrometry currently detects variations.

In addition to the role, studies have shown that ox-LDL can also induce autophagy in endothelial cells, suggesting that autophagy may play a role in the degradation of ox-LDL in endothelial cells [37]. Zhang *et al*. [36] discovered that ox-LDL accumulated in human umbilical vein endothelial cells (HUVECs) and caused an increase in autophagosomes and autophagolysosomes in the cells. The enhancement of ox-LDL-induced autophagy can be inhibited by the Phosphatidylinositol 3-Kinases (PI3K) inhibitor 3-methyladenine and enhanced by the mammalian target of rapamycin (mTOR) inhibitor rapamycin. This suggests that ox-LDL affects the autophagy of endothelial cells through the PI3K/AKT/mTOR signaling pathway (AKT, protein kinase B), and it plays a role in the degradation of ox-LDL.

In addition, ox-LDL contributes to the development of AS from the endothelium to the basement membrane of the arterial wall. It is currently hypothesized that in addition to traversing the endothelial monolayer between adjacent cells by convection and/or diffusion (paracellular leakage), ox-LDL can also enter through active endocytosis of endothelial cells. This endocytosis can be mediated by receptors, such as SR-A and CD36, or it might entirely be liquid-phase endocytosis without receptor mediation [38]. The lipids that infiltrate the vessel wall will induce a variety of cells described below and eventually affect the plaque outcome.

### 5.2 The Complex Lipid Metabolism Mechanism of Macrophage-Derived Foam Cells (MDFCs)

Foam cells, characteristic pathological cells in atherosclerotic plaques, are formed by macrophages or smooth muscle cells phagocytosing a large amount of fat. Foam cells have historically been referred to as MDFCs, unless otherwise noted. MDFCs, which dominate the foam cell line, have garnered increased interest from the academic community due to their related lipid metabolism mechanism.

The accumulation of cholesterol in MDFCs is related to the imbalance of its influx, esterification, and efflux. Scavenger receptors, Class A (SR-A) and CD36 (belonging to the SR class B family) play major roles in cholesterol entry. Both can mediate the uptake of LDL-C by macrophages through phagocytosis and pinocytosis. Upregulation of these receptors via the peroxisome proliferator-activated receptor-γ (PPARγ)-dependent or PPARγ-independent pathway resulted in an increase in foam cells, whereas silencing or downregulation of these receptors had the reverse effect [39].

The esterification and hydrolysis cycle of CE is a crucial component of intracellular cholesterol homeostasis maintenance. After cholesterol is ingested, LDL-C is delivered to late endosomes/lysosomes, and CE is hydrolyzed to FC by lysosomal acid lipase. To prevent FC-related cytotoxicity, the released FC is re-esterified by ACAT on the endoplasmic reticulum and stored in cytoplasmic lipid droplets. If this continues, excess CEs will accumulate in the macrophages and form a “foam”. These resynthesized and stored CEs can be hydrolyzed by neutral cholesterol ester hydrolase (nCEH), to liberate FC for the transporter-mediated efflux, which is increasingly regarded as the rate-limiting step in FC efflux [40].

**Fig. 2.**
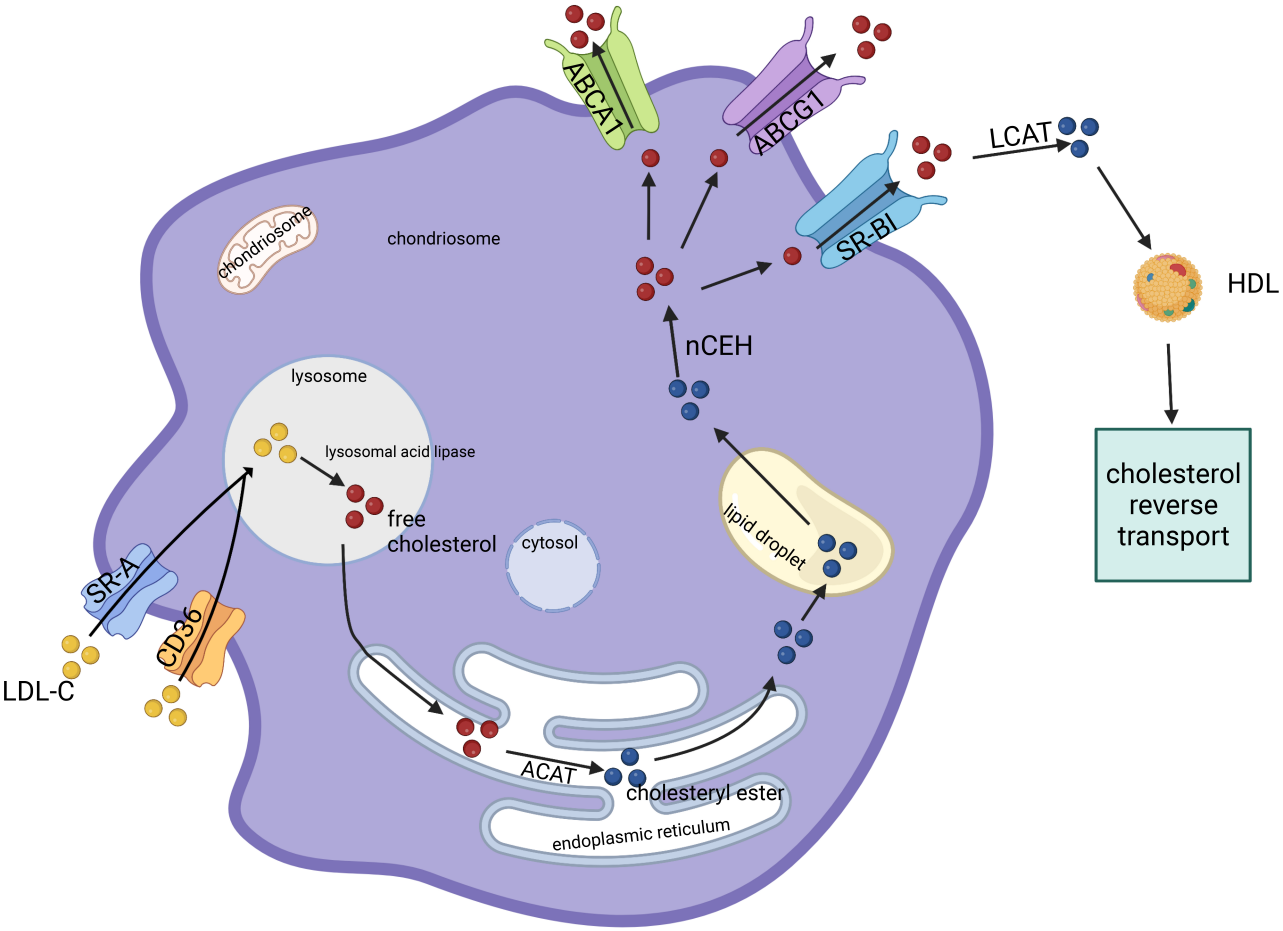
**There is a dynamic equilibrium in the conversion of cholesterol and CEs in MDFCs**. Cholesterol is transported into the cell in the form of LDL-C via SR-A or CD36, and it is decomposed into FC by lysosomal acid lipase in the lysosome. Excessive FC is active and cytotoxic, and it is re-esterified by ACAT in the endoplasmic reticulum to form stable and less toxic CE, which is stored in lipid droplets in the cytoplasm. When CE reserves are very large or there are factors that promote efflux, nCEH can decompose CE into free fatty acids, which are transferred out of MDFCs through ABCA1, ABCG1, SR-BI, and other transporters for secretion into the interstitium. The interstitial LCAT can re-esterify cholesterol. HDL ultimately transports cholesterol in the opposite direction.

FC that exceeds the re-esterification storage capacity of macrophages can be partially effluxed via passive diffusion. Transporters, including as ATP binding cassette transporter A1 (ABCA1), ATP Binding Cassette Transporter, Subfamily G, Member 1 (ABCG1), and scavenger receptor class B type 1 (SR-BI), actively remove a substantial amount of FC from macrophages. If this effluxed FC is collected by high-density lipoprotein (HDL) or apolipoprotein A-I (apoA-I) for reverse cholesterol transport, reversal growth of lipids in plaques will be achieved, which is beneficial to the prognosis of plaques. However, if retrograde transport is not completed, deposition of FC or CE in the ECM will conversely decrease the plaque stability [41] (Fig. 2).

**Fig. 3.**
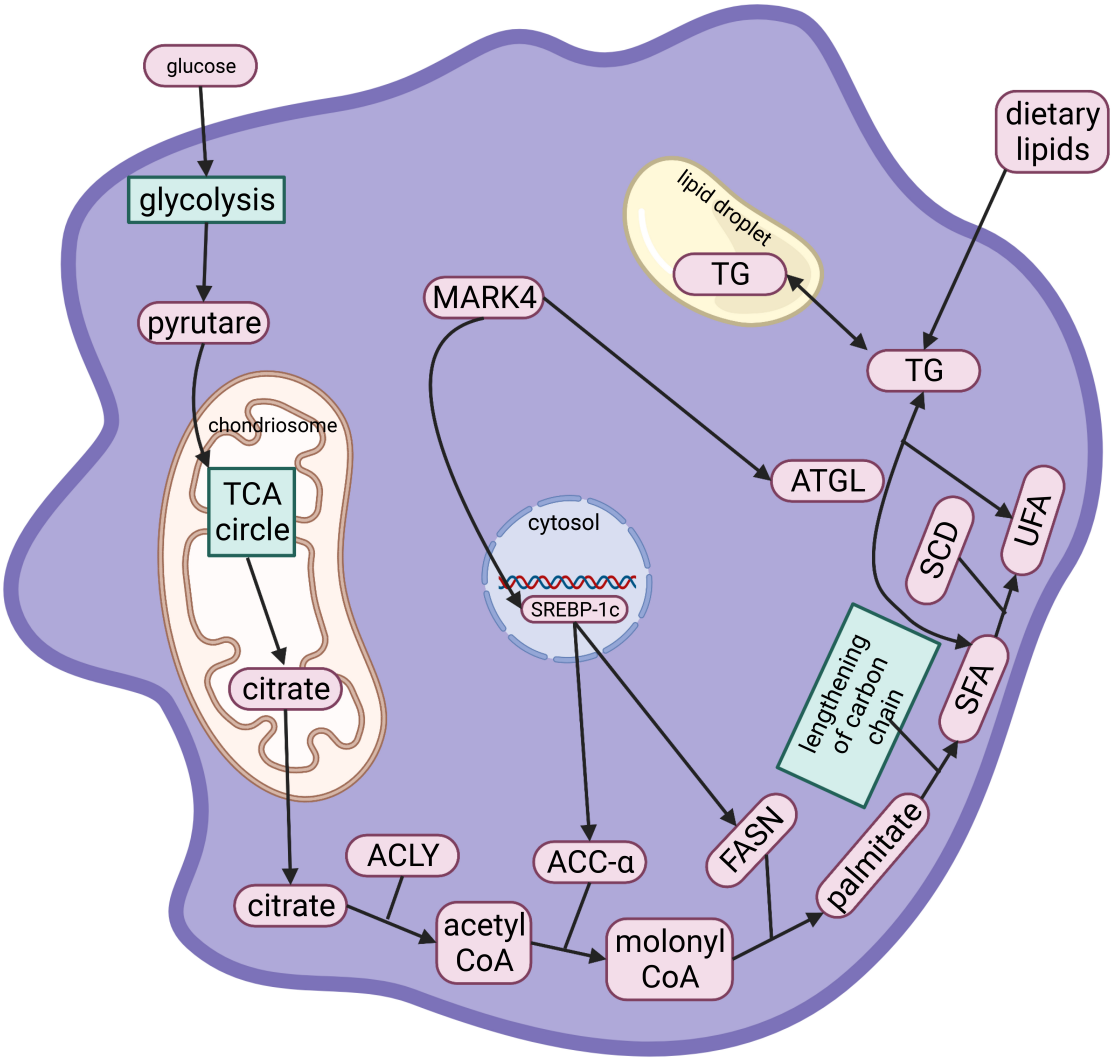
**Metabolism of TG and fatty acids within MDFCs**. The tricarboxylic acid (TCA) cycle and a series of enzymes convert glucose into endogenous fatty acids. Among them, MARK4 regulates the expression of ACC-α and FASN through the transcription factor SREBP-1c, thereby affecting the synthesis of endogenous fatty acids. After TG is synthesized from endogenous or exogenous fatty acids, it is stored in intracellular lipid droplets together with exogenous TG. After fat mobilization, MARK4 is activated, ATGL activity is increased, and TG generates glycerol and fatty acids for energy. TCA, Tricarboxylic acid; ACLY, ATP citrate lyase; ACC-α, acetyl-CoA carboxylase-α; FASN, fatty acid synthase; SCD, stearoyl-CoA desaturase.

The aforementioned lipid absorption process occurs in the form of LDL. PLs and Tgs, which are components of LDL, can also be taken up by MDFCs via the aforementioned scavenger receptors. The difference is that Tgs are employed as energy storage substances, and Tgs and their hydrolyzate fatty acids are frequently generated and decomposed to adapt to the energy metabolism condition of cells. The gene sterol regulatory element-binding protein-1c (SREBP-1c) is considered a major transcription factor for fatty acid biosynthesis [9]. SREBP-1c promotes fatty acid synthesis by enhancing the expression of fatty acid synthase and acetyl-CoA carboxylase alpha (ACCα) at the transcriptional level. MAP-microtubule affinity-regulating kinase 4 (MARK4) can up-regulate the expression of SREBP-1c and ACCα to promote fatty acid accumulation. Adipose triglyceride lipase (ATGL) is a major triglyceride hydrolase in mammals. It has been reported that MARK4 reduces the protein content of ATGL, thereby significantly increasing the accumulation of lipid droplets in cells [42]. Cells achieve internal Tg fatty acid balance by regulating MARK4. The following figure shows how fatty acids affect the development of AS (Fig. 3).

### 5.3 Limited Knowledge about Lipid Metabolism in Smooth Muscle-Derived Foam Cells (SMDFCs)

After undergoing phenotypic transition, smooth muscle cells can function similarly to MDFCs; therefore, they are also called macrophage-like smooth muscle cells. The only difference between the two cell types is their cytomics. After MDFCs become foam cells, studies have not found any their significant differences in the lipid metabolism. Future studies should focus more on how SMDFC lipid metabolism affects the transition process.

Sirtuin1 (SIRT1) is a member of the histone sirtuin family and a mammalian protein homologous to yeast silent information regulator 2 (Sir2). SIRT1 can target many downstream proteins, including PPARγ, PPARγ coactivator-1α, uncoupling protein-2, liver X receptor (LXR), and NF-κB, to affect a wide range of pathophysiological processes. Deacetylation of LXR by SIRT1 upregulates the LXR activity and promotes reverse cholesterol transport to excrete cholesterol from cells, ultimately inhibiting foam cell formation [43]. Inhibition of the aforementioned process by variables such as inflammation will promote the formation of foam cells.

Likewise, inflammation can lead to AS by disrupting the LDL receptor pathway. Subcutaneous injection of lipopolysaccharides in VSMCs induces inflammation, and raises lipid accumulation in the aorta and VSMCs of ApoE Ko mice, as well as the LDL receptor, SREBP cleavage activator protein (SCAP), and SREBP-2, and can enhance the translocation of the SCAP/SREBP-2 complex from the endoplasmic reticulum (ER) to the Golgi apparatus. In addition, inflammation simultaneously increases the percentage of cells in the S phase of the cell cycle and the expression levels of retinoblastoma tumor suppressor protein (Rb), mTOR, eukaryotic initiation factor 4e-binding protein 1 (4EBP1), and phosphorylated forms of P70 S6 kinase. Inflammation alters feedback regulation of the LDL receptor by activating the mTOR pathway. Increased mTORC1 activity upregulates SREBP-2-mediated cholesterol uptake, which induces SMDFC transformation [44]. Inflammation and lipid deposition are mutually reinforcing, generating a vicious cycle that ultimately results in the irreversible transformation of smooth muscle cells into SMDFCs.

## 6. Conclusions

It is vital to maintain a balance between lipid input, metabolism, and plaque release in order to prevent a decline in plaque stability, which can lead to cardiovascular and cerebrovascular events. Increasing evidence suggests that food ingredients play an important role in preventing foam cell formation by reducing cholesterol intake and/or promoting its removal, and reducing SFA intake. Seven phenolic acids, the main bioactive compounds in blueberry, were recently reported to attenuate macrophage foam cell formation by down-regulating the expression of CD36 and up-regulating the expression of ABCA1. Notably, although plasma high-density lipoprotein (HDL) levels are negatively related with the risk of atherosclerotic cardiovascular disease, treatments that raise the HDL level are not always effective. The metabolic preferences of different cells for the same lipids as well as the various metabolic pathways associated with subdivided lipid classes in AS require additional study. The discrepancies shown in studies on plaque lipidomics, as well as the use of existing drugs, such as rapamycin, remind us of the opportunities for early diagnosis and intervention in disease development. In conclusion, further work is necessary to elucidate the distinct processes that regulate these lipid metabolisms and to determine their contribution to protection from human diseases. These studies will provide additional insights into the physiopathological roles of atherosclerotic cardiovascular disease and reveal new therapeutic strategies for the treatment of atherosclerotic cardiovascular disease.
